# Aptamer-conjugated graphene oxide-based surface assisted laser desorption ionization mass spectrometry for selective extraction and detection of Aβ_1–42_ in an Alzheimer’s disease SH-SY5 cell model

**DOI:** 10.3389/fnagi.2022.993281

**Published:** 2022-09-20

**Authors:** Gongshuai Song, Ruofan Shui, Danli Wang, Ruosi Fang, Tinglan Yuan, Ling Li, Junli Feng, Feng Gao, Qing Shen, Jinyan Gong, Fuping Zheng, Manman Zhang

**Affiliations:** ^1^Zhejiang Provincial Key Lab for Biological and Chemical Processing Technologies of Farm Product, School of Biological and Chemical Engineering, Zhejiang University of Science and Technology, Hangzhou, China; ^2^Zhejiang Province Joint Key Laboratory of Aquatic Products Processing, Collaborative Innovation Center of Seafood Deep Processing, Institute of Seafood, Zhejiang Gongshang University, Hangzhou, China; ^3^Hangzhou Linping Hospital of Traditional Chinese Medicine, Hangzhou, China; ^4^Beijing Laboratory of Food Quality and Safety/Key Laboratory of Alcoholic Beverages Quality and Safety of China Light Industry, Beijing Technology and Business University, Beijing, China; ^5^Department of Neurology, The First Affiliated Hospital of Wenzhou Medical University, Wenzhou, China

**Keywords:** amyloid beta-peptide Aβ_1–42_, graphene oxide, aptasensor, selective enhancement, SALDI-MS

## Abstract

The generation and accumulation of amyloid-beta peptide (Aβ_1–42_) in amyloid plaques are key characteristics of Alzheimer’s disease (AD); thus, specific detection of Aβ_1–42_ is essential for the diagnosis and treatment of AD. Herein, an aptamer-conjugated graphene oxide (Apt-GO) sensor was synthesized by π-π and hydrophobic interactions using thiol poly (ethylene glycol) amine (SH-PEG-NH_2_) as a spacer unit. Then, it was applied to selective capture of Aβ_1–42_, and the resulting complex was directly analyzed by surface-assisted laser desorption ionization mass spectrometry (SALDI-MS). The results revealed that the Apt-GO could enhance the detection specificity and reduce non-specific adsorption. This method was validated to be sensitive in detecting Aβ_1–42_ at a low level in human serum (ca. 0.1 μM) within a linear range from 0.1 to 10 μM. The immobilizing amount of aptamer on the GO was calculated to be 36.1 nmol/mg (RSD = 11.5%). In conclusion, this Apt-GO-based SALDI-MS method was sensitive and efficient in selective extraction and detection of Aβ_1–42_, which proved to be a good option for early AD diagnosis.

## Introduction

Alzheimer’s disease (AD) appears to be the most prevalent neurodegenerative disorder specified by cognitive impairment, progressive memory, and behavioral function and language deterioration, which affect economic, professional, social, and personal functions (Zamanian et al., 2022; Zheng et al., 2022). Nowadays, the number of AD patients is expected to reach 36 million people and further increase to 152 million people worldwide in 2050 (Zhang et al., 2021). Nowadays, the clinical diagnosis of AD mainly depends on neuroimaging and psychological tests. However, the cost of neuroimaging is high and the accuracy of cognitive evaluation is low, which was apt to cause misdiagnosis and delay the treatment of patients with AD. The pathological characteristic of AD is the formation of neurofibrillary tangles and the accumulation of amyloid plaques because of highly phosphorylated microtubule-related Tau protein filaments. It was demonstrated that amyloid beta-peptide (Aβ) is a potential biomarker for AD diagnosis. The key component of Aβ aggregates is the Aβ_1–42_ (Hamd-Ghadareh et al., 2022). It is essential for the development of a rapid and reliable specific enrichment and detection technique based on Aβ for the early diagnosis of AD.

To date, various analytical methods have been established for detecting Aβ_1–42_, mainly including enzyme-linked immunosorbent assay (ELISA), surface-enhanced Raman spectroscopy, surface plasmon resonance, and electrochemical method (Xia et al., 2016a,^2017^). These methods have been extensively applied in a lab to monitor Aβ_1–42_; however, a typical sandwiched ELISA is labor- and time-consuming due to cross-reactivity (Xia et al., 2016b; Ma et al., 2022; Yang et al., 2022). In contrast, the innovative electrochemical method for the detection of Aβ monomers and oligomers is relatively simple and sensitive. Surface-assisted laser desorption ionization (SALDI) is a soft ionization technology that uses a laser energy-absorbing matrix to create ions with minimal fragmentation based on different nanostructured substrates (Guinan et al., 2015). It has displayed great superiority considering its fast analysis of analytes in seconds, simple sample preparation for broad application, the accurate mass measurement for identification, and high sensitivity with low costs for monitoring disease biomarkers from biological samples (Huang et al., 2017). The existence of Aβ_1–42_ is at ultra-trace levels and a large number of impurities and interfering proteins were mixed in human body fluids, which caused a strong challenge for the direct analysis of Aβ_1–42_. Hence, selective enrichment of Aβ_1–42_ is highly crucial for in-depth pathological research of AD before SALDI-MS analysis.

Recently, biosensors, such as aptamer-based sensing devices, have played an important role in the detection of Aβ_1–42_. Aptamers are synthetic single-stranded RNA or DHA sequences that are obtained by systematic evolution of ligands employing exponential enrichment (SELEX) technology, which can bind to their targets with high affinity and specificity (Huang et al., 2021). It revealed that aptamers have numerous advantages, including biocompatibility, simplicity in synthesis, less immunogenicity, lack of cross-reactivity, long shelf life, and resistance to high temperature and chemical stress (Díaz-Fernández et al., 2021). Penner et al. (2021) developed the aptamer-based biosensor for Aβ prediction on the basis of the aptamer library election. It reported that graphene oxide (GO)-based aptasensors have been developed because of the large surface area and strong adsorption ability of GO. Deng et al. (2016) achieved the specific detection of human α-thrombin using aptamer-conjugated gold functionalized GO nanocomposites.

In this study, a novel biosensor was developed by conjugating Aβ_1–42_-specific aptamer on GO, and the aptamer-conjugated GO (Apt-GO) play the role in selective enrichment and SALDI-MS detection of Aβ_1–42_.

## Materials and methods

### Chemicals and materials

Graphite powder with a purity of 99.95% and Aβ_1–42_ peptides were purchased from Sigma-Aldrich (St. Louis, MO, USA). Trifluoroacetic acid (TFA), magnesium chloride (MgCl_2_), sodium nitrate (NaNO_3_), sulfuric acid (H_2_SO_4_), hydrogen peroxide (H_2_O_2_), potassium permanganate (KMnO_4_), acetonitrile (ACN), TFA, sodium chloride (NaCl), thiol poly(ethylene glycol) amine (SH-PEG-NH_2_, purity ≥ 95%), N-hydroxysuccinimide (NHS, purity ≥ 98%), and 1-ethyl-3-(3-dimethylaminopropyl)carbodiimide (EDC, purity ≥ 99%) were obtained from Merck (Darmstadt, Germany). High purity water (18.2 MΩ⋅cm) was filtered by a Milli-Q system (Millipore, Bedford, MA, USA). The other chemicals and solvents of analytical grade were obtained from Xilong Scientific Co., Ltd. (Guangzhou, China). The SH-SY5Y cell line was obtained from BeNa Culture Collection (Beijing, China). Dulbecco’s modified essential medium (DMEM), fetal bovine serum (FBS), penicillin, streptomycin, all-trans retinoic acid (ATRA), and brain-derived neurotrophic factor (BDNF) were purchased from Gibco (SanFrancisco, CA, USA). Human serum samples were provided by Linping Hospital of Traditional Chinese Medicine (Hangzhou, China).

The aptamer for Aβ_1–42_ (5′-AGT CTA GGA TTC GGC GTG GGT TAA TTT TTT GCT GCC TGT GGT GTT GGG GCG GGT GCG-3′) was previously reported and synthesized by Sangon Biotechnology Inc. (Shanghai, China) (Chan et al., 2019). It was modified with a 5′-thiol modifier for GO modification and further labeled with fluorescein at the 3′-end for detecting and quantifying the modification on the GO surface.

### Synthesis of graphene oxide

GO was synthesized according to a modified Hummer’s method (Zhao et al., 2020; Song et al., 2021). In brief, 2 g of graphite powder and 1 g of NaNO_3_ were dissolved in 40 mL H_2_SO_4_ in a round bottle flask in an ice bath, because the reaction could produce a slight exotherm. After cooling to room temperature (ca. 25°C), the ice bath was removed and the reaction was sustained for 30 min after the gradual addition of 6 g KMnO_4_ under stirring. Then, 30% H_2_O_2_ solution (20 mL) was gradually added to remove excess KMnO_4_ until the mixture turned brilliant yellow. Afterward, the synthesized GO mixture was neutralized using 5% HCl and ultrapure deionized water after centrifugation (10,000 *g* for 15 min at 4°C). The residual salt in the obtained solid material was removed by dialysis in deionized water. Finally, the synthesized GO was dried under vacuum at room temperature overnight and characterized by a scanning electron microscope (SEM, FEI Inspect F50, Thermo Fisher Scientific, MA, USA).

### Construction of sensor

**FIGURE 1:**
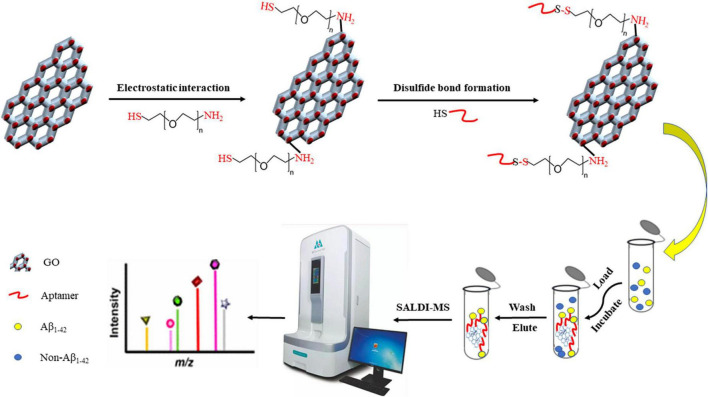
Procedure of the aptamer-modified GO nanoparticles fabrication and application for detecting Aβ_1–42_ coupled with SALDI MS.

The procedure for fabricating and utilizing Apt-GO for detecting Aβ_1–42_ under SADLI MS was displayed in Figure 1. According to the method described in Gulbakan et al. (2010) with slight modifications, the PEGylation of GO was performed (Gulbakan et al., 2010). In brief, the GO solution (1 mg/mL) was dispersed in PBS buffer (pH = 7.4) containing EDC (0.0022 M) and NHS (0.0015 M), followed by sonicating for 8 h to activate the carboxyl groups. Afterward, HS-PEG-NH_2_ (0.1306 M) was added to the solution and the mixture was sonicated for 1 h at room temperature. By centrifugation at 10,000 *g* for 10 min four times, the excess ligand was washed away. The precipitate was redispersed in 100 μL of PBS (pH = 7.4). The aptamer solution was heated at 90°C for 30 min, followed by cooling at room temperature. The obtained PEGylated GO (100 μL) was incubated with 5′-SH-Aptamer in PBS (200 μL). Then, the mixed solution was centrifuged at 10,000 *g* for 10 min and washed several times to remove the excess unbound aptamers. The precipitate was dispersed in 300 μL ultrapure water.

### Preparation of Aβ_1–42_

The preparation of Aβ_1–42_ was carried out by using SH-SY5Y neuroblastoma cells as AD model cells according to the previous study (Feng et al., 2021). The SH-SY5Y cell line was cultured in DMEM. Before experiments, the SH-SY5Y cells were grown for three generations and used at a low passage number (<13). In detail, the cells were cultured in a humidified incubator at 37°C with 5% CO_2_. The culture medium was supplemented with 10% heat-inactivated FBS and 100 U/mL penicillin/streptomycin, and it was replaced three times per week. After reaching 70–80% confluence, the cells were differentiated with ATRA, DMEM, and BDNF in the medium. The treated cells with neuron-like phenotype were processed with Aβ_1–42_ for 24 h and harvested as AD models.

### Surface-assisted laser desorption ionization-TOF/mass spectrometry analysis

During the detection of Aβ_1–42_, GO played the role of matrix in SALDI-MS analysis. GO (1 mg) was dispersed in ultrapure water (1 mL) under sonication for 20 min. The above GO slurry (2 μL) was successively pipetted on the stainless steel target plate and dried for SALDI-MS analysis. Based on the reflection mode employing delayed extraction, the MS analysis was performed on 5800 Proteomics Analyzer (Applied Biosystems, Framingham, MA, USA) coupled with a pulsed Nd:YAG laser (1 kHz, 355 nm wavelength). In this study, the delay time, acceleration voltage, and repetition rate were optimized to 200 ns, 20 kV, and 200 Hz, respectively. For the SALDI-MS analysis, the number of laser shots was 200 per analysis.

For selective enhancement of Aβ_1–42_, the Apt-GO slurry was centrifuged at 4,000 *g* for 15 min and the precipitate was redispersed in the binding buffer (20 mM Tris-HCl, 140 mM NaCl, and 2 mM MgCl_2_, pH = 7.5). Then, the Aβ_1–42_ standards and disrupted AD cells model solution were added. The mixture was incubated at 37°C for 30 min and centrifugated at 4,000 *g* for 15 min. Afterward, the precipitate was redispersed in 0.1% TFA solution for SALDI-TOF/MS analysis as described above.

### Statistical analysis

The MS data were processed by the Data Explore Software called Analyst (AB Sciex, USA). Statistical analysis was carried out by Microsoft office software, IBM SPSS Statistics (version 21.0), and the analysis of variance (ANOVA) followed by Duncan’s new multiple range tests. All experiments above were performed at least in triplicate.

## Results and discussion

### Characterization of aptamer-conjugated graphene oxide

**FIGURE 2:**
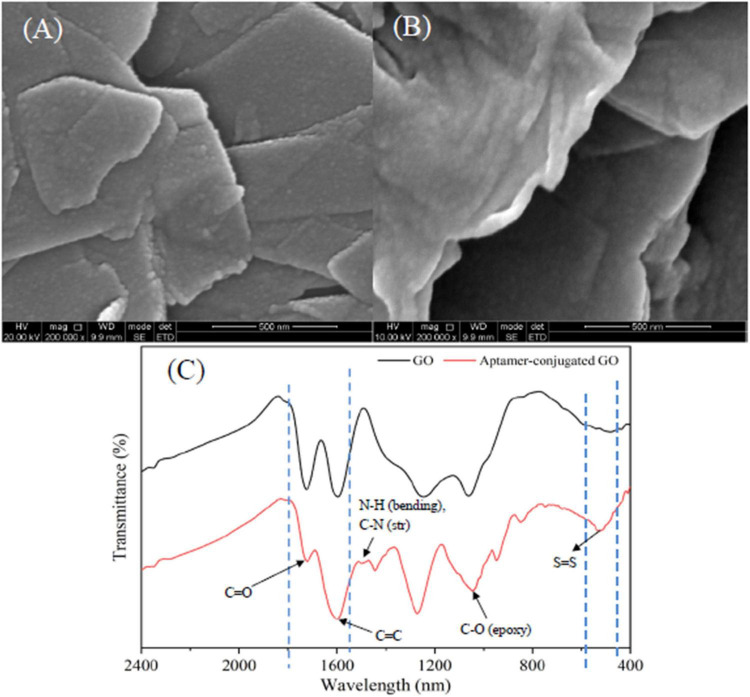
SEM micrographs of graphite **(A)** and GO **(B)**; FT-IR spectra of GO and aptamer-modified GO nanoparticles **(C)**.

The morphology and surface of graphite and GO were characterized by a scanning electron microscope (SEM, FEI Inspect F50, Thermo Fisher Scientific, MA, USA). As seen in Figure 2A, the interlayer space of graphite was small and the surface was relatively smooth. After chemical modification (Figure 2B), the rough surface and larger lamellar structure were formed with many wrinkles due to the presence of oxygen-containing functional groups. It was reported that the surface of GO contains many polar moieties, mainly including hydroxyl epoxy and carboxy groups, which ensures that the GO has more polar and hydrophilic characteristics (Du et al., 2020).

Typically, PEGylation of GO nanoparticles is achieved by a ligand exchange process using HS-PEG-NH_2_ molecules as a spacer unit. In this study, the bifunctional PEG linker carriers, –NH_2_ groups, bind to the carboxyl groups on the surface of GO nanoparticles, and the –SH groups attach to the SH-functionalized aptamers by forming a disulfide bond (Xia et al., 2012). According to Fourier transform infrared (FTIR) spectrum depicted in Figure 2C, the characteristic peaks of GO nanoparticles at 1,720 and 1,610 cm^–1^ were carboxyl (C = O) stretching and vibration of carbon skeleton (C = C), respectively. The characteristic peak of the symmetric tensile vibration of the epoxy group (C-O) was evident at 1,050 cm^–1^. The result was in line with the previous reports (Du et al., 2020), which further demonstrated that GO nanoparticles were fabricated successfully. In addition, the FTIR spectrum of aptamer-modified GO was characterized. The N-H bending and C-N stretching vibration peaks were observed in the aptamer-modified GO nanoparticles at 1,535 cm^–1^, indicating the conjugation of HS-PEG-NH_2_ with GO. This is attributed to the exposure to amine groups of GO resulting in nucleophilic attacks (Caicedo et al., 2020). The characteristic peak of a disulfide (S-S) bond was observed at 540 cm^–1^, representing the cross-linking of SH-functionalized aptamers and the thiol groups in the HS-PEG-NH_2_ molecules. The above result verified that the conjunction between aptamers and GO nanoparticles using HS-PEG-NH_2_ molecules was successful.

The aptamer binding capacity of the Apt-GO nanoparticles was evaluated by comparing the UV adsorption value of the supernatant before and after immobilization at the wavelength of 260 nm. The binding capacity was calculated to 36.1 nmol/mg (RSD = 11.5%, *n* = 3), much higher than that of GO (50 pmol/mg). The higher immobilized amount should be attributed to the ultra-high specific surface area of PEGylation of GO nanoparticles.

### Performance of aptamer-conjugated graphene oxide sensor

**FIGURE 3:**
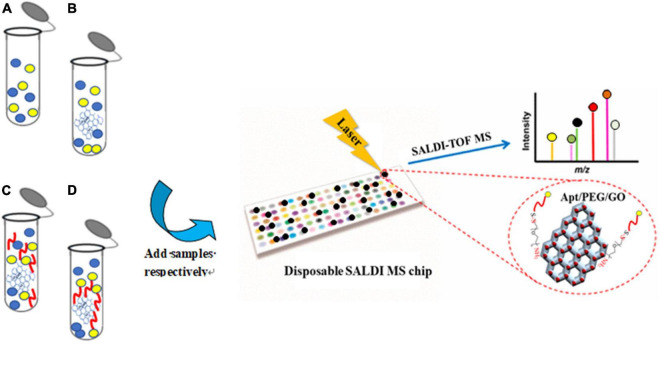
Schematic analysis of the GO-based aptamer-assisted mass spectrometry analysis. **(A)** Analysis of analyte ionized on GO without extraction; **(B)** analysis of analyte extracted with GO and ionized on GO; **(C)** analysis of analyte extracted with non-covalent-aptamer bound GO and ionized on GO; **(D)** analysis of analyte extracted with covalent-aptamer bound GO and ionized on GO.

To evaluate the selectivity of the Apt-GO sensor, a schematic analysis of the GO-based aptamer-assisted MS analysis was carried out. As seen in Figure 3, four replication experiments were designed. Group A was used to prove the function of GO as the matrix for ionization on SALDI MS analysis. The GO solution was spotted on the SALDI plate and dried under room temperature; then, the analyte was spotted on the GO matrix and dried for laser desorption ionization. Group B was used to analyze the analyte extracted with GO and ionized on GO. The GO was added to the analyte solution for extraction. Afterward, the mixture was washed rigorously, and the supernatant was removed. The obtained GO pellets were spotted on the SALDI plate for ionization analysis. Group C was used to analyze the analyte extracted with non-covalent-aptamer bound GO. The mixture of the aptamer and GO solution was added to the analyte for selective enrichment. Group D was designed to evaluate the selectivity of the Apt-GO by covalent bonding. The aptamer-modified GO was added into the analyte for specific enrichment. The following steps were the same as in the above experiments.

**FIGURE 4:**
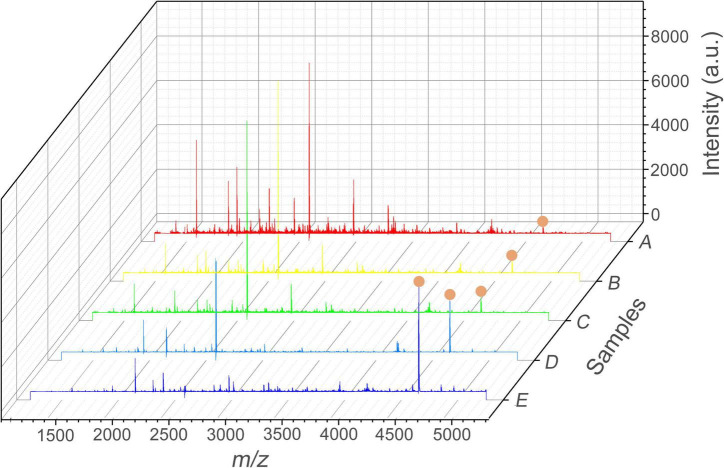
SALDI MS spectra of Aβ_1–42_ ([M + H]^+^, m/z = 4515.7) in the AD cell model. **(A)** Normal group without aptamer modification and any extraction step; **(B)** test group extracted with GO; **(C)** test group extracted with aptamer-modified GO non-covalently; **(D)** test group extracted with aptamer-modified GO covalently; **(E)** MS spectrum of Aβ_1–42_ standard spiked in the human serum sample. Mass spectrometric peaks of Aβ_1–42_ were labeled with red circles.

The performance of the Apt-GO was characterized by SALDI MS. Figure 4A shows the MS analysis of Aβ_1–42_ in the AD cell model analyzed on GO served as the matrix without aptamer modification and any extraction step. Under the positive ionization mode, the SALDI-MS of the Aβ_1–42_ could be detected in *m/z* of 4,515.7 with an average signal-to-noise (S/N) value of 15 among huge background ions. The analysis result of the AD cell model containing Aβ_1–42_ enriched and ionized with GO nanoparticles without modification is displayed in Figure 4B. The peak intensity and S/N value were enhanced slightly, and the background ions were reduced. This was mainly attributed to the unique morphology of GO nanoparticles that could facilitate the diffusion of Aβ_1–42_, which was beneficial for the extraction of Aβ_1–42_ from complex biological samples. In addition, it was found that the π-π and hydrophobic interactions existed between GO nanoparticles and Aβ_1–42_ peptide, which could further stabilize the cross-linking between GO nanoparticles and Aβ_1–42_ by salt bridges and H-bonds (Sun et al., 2016; Jin et al., 2019). As seen in Figure 4C, the peak intensity of Aβ_1–42_ was low and the S/N value of 17 was obtained. This result could be attributed to non-covalent-aptamer bound GO. During the washing step, the aptamer-captured parts of Aβ_1–42_ were released from the surface of GO due to the absence of chemical functionalization. However, the background of MS was relatively clear to some extent, which could be ascribed to the physical adsorption capacity of GO (Sun et al., 2008). In this study, the MS signal was generated from the surface of GO. After the washing step, the peak signal of Aβ_1–42_ decreased because of the removal of the aptamer-captured Aβ_1–42_. On the contrary, the peak intensity and S/N value of Aβ_1–42_ were both enhanced significantly (*p* < 0.05) due to the covalent modification between GO and the aptamer by chemical conjugation (Figure 4D). Figure 4E showed the peak of Aβ-42 standard spiked in human serum sample, which was used for comparison. This result demonstrated that the performance of Apt-GO for selective enhancement of Aβ_1–42_ was efficient, and chemical conjugation of the aptamer was critical for analyte capture. This result was in line with the reported study for GO-based fluorescence sensors. It revealed the restoration of fluorescence that had previously been quenched by GO after the aptamer left the surface upon target addition (Wang et al., 2010).

### Recognition reproducibility of aptamer-conjugated graphene oxide sensor

It is necessary to evaluate the recognition reproducibility of Apt-GO for capturing target protein. In this study, one batch of Apt-GO was fabricated and stored at 4°C every week. A total of five batches of the sensors were fabricated within 5 weeks. All obtained sensors were incubated with Aβ_1–42_ at the same time after the final batch was fabricated. According to the SALDI-MS result, the Aβ_1–42_ could be captured by Apt-GO fabricated in different weeks with similar peak intensities and S/N values (RSD, 15.2%). This result was attributed to the stable covalent bond between GO and aptamers by the π-π and hydrophobic interactions, which exhibited enhanced resistance to nucleases and protected aptamer from enzymatic digestion effectively (Tang et al., 2012).

### Reusability of aptamer-conjugated graphene oxide sensor

**FIGURE 5:**
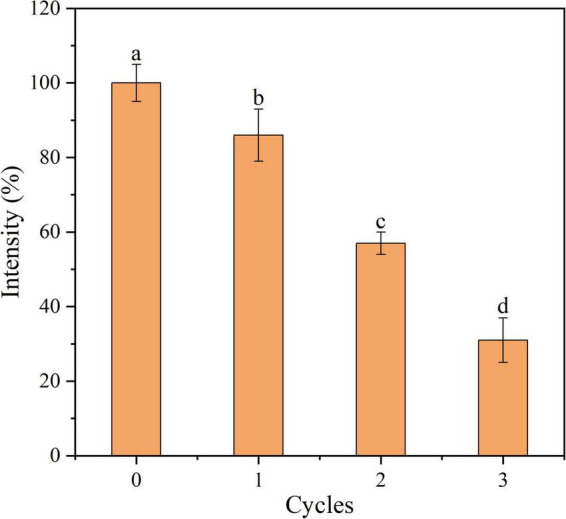
Recycled use of aptamer-modified GO nanoparticles based sensor for Aβ_1–42_ adsorption.

The reusability of Apt-GO for target proteins is essential in practical applications. It reveals that the conformation between aptamer and target protein is formed and maintained by the intra-molecular forces, mainly including hydrogen bonding and hydrophobic and van der Waals interactions. Once this conformation changes, the binding affinity between the aptamer and its target will disappear (Ocsoy et al., 2013). In theory, aptamers undergo conformational changes upon thermal denaturation. In this study, the Aβ_1–42_ was released from Apt-GO by heating. The Apt-GO could be reused after centrifugation and washing procedures. The reusability of Apt-GO for Aβ_1–42_ was carried out at 65°C, and the result revealed that the enrichment efficiency was nearly 70% loss after three extraction cycles (Figure 5). This result was mainly ascribed to the destroyed conformation of aptamers and broken chemical conjugation between GO and aptamers caused by thermal denaturation. This result indicated that the enrichment performance of recycled Apt-GO would degenerate significantly (*p* < 0.05).

### Real samples analysis

To further investigate the feasibility and practical applicability of Apt-GO in the measurement of Aβ_1–42_, human blank serum samples with different concentrations of Aβ_1–42_ were prepared (0.1, 1, and 10 μM). Under the same condition, the prepared samples were incubated with Apt-GO independently. As seen in Table 1, the recoveries of samples were in the range of 95.10–101.28%, indicating superior detection performance, dependability, and accuracy in real samples. Although the data for the three samples were limited, the work demonstrated that it was possible to distinguish patients from healthy people by improving the sensitivity of the biosensor for AD biomarkers.

**TABLE 1:** Detection of the proposed sensor for capture of Aβ_1–42_ in serum samples.

Samples	Added (nM)	Found (nM)	Recovery (%)	RSD (%, *n* = 3)
1	0.1	0.0951 ± 0.0023	95.10	10.5
2	1.0	1.0128 ± 0.3677	101.28	8.2
3	10.0	9.8766 ± 0.0715	98.77	12.6

## Conclusion

In summary, the Apt-GO sensors were fabricated and applied for selective enrichment and detection of Aβ_1–42_ from disrupted AD cells model solution based on SALDI MS. The results demonstrated that the proposed aptasensor eliminated the need for any additional energy-absorbing matrix for ionization and reduced background interference in SALDI matrices. The large surface area, excellent hydrophilicity, and good biocompatibility of the aptasensor ensure the advantages of high sensitivity and selectivity for selective enrichment of target analytes and the attainment of direct mass spectrometric readouts. This study provides a simple, applicable, highly selective and sensitive, and rapid (treatment time of ca. 15 min) SALDI MS method to detect the low concentrations of Aβ_1–42_, which offers promising guidance for developing novel enrichment aptasensor combined with SALDI MS for the disease biomarker detection and clinical diagnosis at the early stage of the disease.

## Data availability statement

The raw data supporting the conclusions of this article will be made available by the authors, without undue reservation.

## Ethics statement

There were no studies on human participants contained in this article. All applicable guidelines followed for the use and care of animals were institutional, national, and international.

## Author contributions

GS: roles/writing—original draft. RS, JF, FG, and FZ: formal analysis, validation, and interpretation of the data. DW: formal validation, formal analysis, and interpretation of the data. RF: validation, data curation, and formal analysis. TY: methodology, validation, and formal analysis. LL: formal validation and interpretation of the data. QS: funding acquisition, formal analysis, and validation. JG: conceptualization, validation, and analysis. MZ: data curation, validation, and analysis. All authors contributed to the article and approved the submitted version.
